# Facteurs prédictifs de l’issue de l’accouchement sur utérus unicicatriciel, expérience du centre de Maternité de Bizerte

**DOI:** 10.11604/pamj.2016.25.76.9164

**Published:** 2016-10-10

**Authors:** Amira Ayachi, Sadok Derouich, Insaf Morjene, Lassaad Mkaouer, Dalila Mnaser, Mechaal Mourali

**Affiliations:** 1 Université El Manar2, Tunis, Tunisie; 2 Faculté de Médecine de Tunis, Tunisie; 3 Service de Gynécologie et Obstétrique, CHU Bougatfa, Bizerte, Tunisie

**Keywords:** Trial of labor, previous cesarean section, vaginal birth after cesarean section, uterine rupture, scarred uterus, Trial of labor, cesarean section, previous cesarean section, vaginal birth after cesarean section, scarred uterus

## Abstract

**Introduction:**

L’incidence des utérus cicatriciels est en nette augmentation ces dernières décennies. L’obstétricien est alors fréquemment sollicité à décider du mode d’accouchement le plus approprié à la mère et son fœtus. L’objectif de cette étude est de décrire la pratique obstétricale d’un accouchement sur utérus unicicatriciel au sein de notre service et d’identifier les facteurs significativement associés à l’échec de la tentative de voie basse après césarienne.

**Méthodes:**

Nous avons mené notre étude sur une population de femmes porteuses d’un utérus unicicatriciel. Elle était rétrospective longitudinale de type descriptif et analytique. Nous l’avons réalisée sur une période de deux ans et trois mois du 1^er^ Janvier 2012 au 31 Mars 2014 durant laquelle nous avons colligé 423 dossiers de patientes qui ont eu une tentative de voie basse au centre de maternité et de néonatologie de Bizerte.

**Résultats:**

Le taux de tentatives de voie basse après césarienne était de 47%. Les taux de succès et d’échec de ces tentatives étaient respectivement de 82,7% et 17,3%. Les principaux facteurs de mauvais pronostic de la tentative de voie basse après césarienne étaient: l’absence d’antécédent d'accouchement par voie basse (p=0,005), une indication de la césarienne antérieure pour stagnation de la dilatation ou défaut d’engagement (p respectifs de 0,049 et 0,002), un terme d’accouchement? 40 SA (p=0,046), une parité < 3 (p=0,75.10-4), un score de Bishop <6 au début du travail (p=0,23.10-47), une phase active de durée? 6h (p=0,002), un travail de durée >8h (p=0,0031) et la survenue d’une anomalie du RCF au cours du travail (p=0,144.10-9). Nous avons observé sept cas de rupture utérine soit un taux de 1,7%. Nous n’avons observé aucun cas de mortalité maternelle. La morbidité maternelle globale était de 9,5%. La différence des taux de complications maternelles entre les deux groupes échec et succès de la tentative de voie basse après césarienne n’était pas statistiquement significative.

**Conclusion:**

La tentative de voie basse après césarienne décidée après revue des facteurs de bon et de mauvais pronostic et consentement de la patiente, contribue à la diminution de la morbidité maternelle et fœtale et devrait aboutir à l’établissement de recommandations tunisiennes claires et codifiées, entrant dans le cadre d’une politique de lutte contre les césariennes itératives non justifiées.

## Abstract (en)

**Introduction:**

Caesarean section (CS) rates have been significantly increasing in recent decades. For this reason, the obstetrician must frequently decide on the most appropriate mode of delivery for mother and fetus. This study aims to describe vaginal birth after previous cesarean section (VBACs) in our obstetric practice and to identify factors significantly associated with failed VBACs.

**Methods:**

We conducted a population-based study among women with a history of previous cesarean delivery. The study design was retrospective, longitudinal, descriptive and analytical. The case study was conducted over a two years and three months period, from January 1, 2012 to March 31, 2014 during which we collected data from 423 medical records of patients attempting VBACs at the Maternity and Neonatology Center, Bizerte.

**Results:**

The rate of attempted VBACs was 47%. The success and the failure rates of these attempts were 82,7% and 17,3% respectively. The main factors for a poor prognosis in patients attempting VBACs were: the absence of a previous vaginal delivery (p = 0.005), a previous indication for cesarean section due to stagnation of dilatation or poor labor progress, (p 0.049 and 0.002 respectively), gestational age at delivery of = 40 weeks (p = 0.046), parity <3 (p = 0,75.10-4), Bishop score <6 at the onset of labor (p = 0,23.10-47), “active labor” duration = 6h (p = 0.002), length of labor> 8 h (p = 0.0031) and the occurrence of abnormal fetal heart rate (FHR) during labor (p = 0144.10 -9). We observed seven cases of uterine rupture (1.7%). There were no cases of maternal mortality. Total maternal morbidity rate was 9,5%. The difference in rates of maternal complications between the two groups (failed and successful attempted vaginal birth after cesarean) was not statistically significant.

**Conclusion:**

Attempting vaginal birth after cesarean on the basis of good and poor prognostic factors and patient consent, contributes to the reduction in maternal and neonatal morbidity and should lead to the establishment of clear and codified Tunisian guidelines as part of a policy against unjustified iterative caesarean sections.

## Introduction

L’accouchement par voie basse est la voie naturelle qui permet une moindre morbidité pour la mère et le nouveau-né [1]. En effet, même si le taux de mortalité maternelle imputable à la césarienne (CS) est faible (3 pour 10 000), il reste 2 à 4 fois supérieur à celui de l´accouchement par voie vaginale [2].

En Tunisie ce taux n’a cessé d’augmenter depuis 1995 où il a été estimé à 12,8% [3] pour atteindre les 20,5% entre 2001 et 2006 [4] puis les 26,7 % entre 2011 et 2012 [5]. Devant le nombre alarmant du taux de césariennes, notamment sur utérus cicatriciel, le mythe « une césarienne un jour une césarienne toujours » devient une réalité. La connaissance des facteurs de risque d’échec de l’épreuve utérine est primordiale avant de décider du mode d’accouchement sur un utérus cicatriciel. Encourir un travail laborieux, pour avoir enfin recours en urgence à une césarienne non programmée et non attendue par la patiente, pourrait l’exposer à de multiples complications [6] ainsi qu’à un traumatisme psychologique et physique [3].

L’objectif de notre travail était de décrire la pratique obstétricale d’un accouchement sur utérus uni cicatriciel au sein de notre service et d’identifier les facteurs significativement associés à l’échec de l’épreuve utérine, pour éviter ainsi de multiples complications [6] ainsi qu’un traumatisme psychologique et physique [3].

## Méthodes

Il s’agit d’une étude rétrospective longitudinale descriptive et analytique sur une période de deux ans et trois mois allant du 1er Janvier 2012 au 31 Mars 2014 réalisée au centre hospitalo-universitaire de gynécologie-obstétrique et de néonatologie de Bizerte. Le recueil des données a été fait à travers les dossiers archivés au service de gynécologie et obstétrique. La population étudiée et qui a constitué notre base de sondage est formée par l’ensemble des patientes porteuses d’un utérus unicicatriciel qui ont eu une épreuve utérine durant la période de notre étude ou tentative d’accouchement par voie basse après césarienne (TVBAC).

Ont été incluses les patientes porteuses d’un utérus unicicatriciel d’origine obstétricale, avec non persistance de l’indication de la première césarienne, un bon bassin osseux, un fœtus unique, une présentation céphalique, une entrée spontanée en travail, avec possibilité d’assurer une surveillance maternelle et fœtale au cours du travail et en post-partum. Ont été exclus les dossiers comportant des signes de souffrance fœtale aigue, une insertion aberrante du placenta, la présence d’un état pathologique maternel nécessitant une évacuation utérine urgente. Nous n’avons pas considéré le poids fœtal estimé par échographie supérieur à 4 kg comme étant un critère d’exclusion. Nous avons utilisé le logiciel SPSS pour les calculs et moyennant les tests statistiques suivants: Chi-square test, test exact de Fischer et le T-Test.

## Résultats

**Figure 1:**
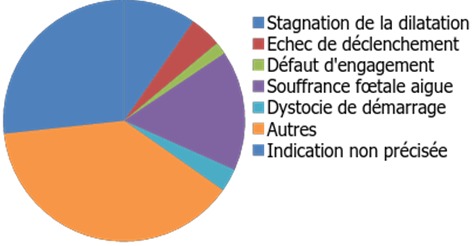
Les différentes indications de la première césarienne chez les parturientes avec utérus unicicatriciel

Au cours de notre étude nous avons recensé 9991 accouchements et 423 dossiers de patientes ayant eu une épreuve utérine soit un taux de 4%. 350 parturientes ont accouché par voie basse et 73 parturientes ont accouché par césarienne en urgence au cours de cette épreuve. L’âge moyen des parturientes de notre échantillon était de 31,61 ans avec des extrêmes de 18 et 45 ans. La répartition des parturientes selon la présence ou non d’un antécédent d’accouchement par voie basse après la césarienne est décrite dans le Tableau 1. L’indication de la première césarienne est spécifiée dans la Figure 1. Nos parturientes ont toutes subi une tentative de voie basse après césarienne et elles ont été réparties en deux groupes selon l’issue de cette tentative : On désigne par le groupe 1, l’ensemble des candidates avec succès de la TVBAC, On désigne par le groupe 2, les candidates dont la TVBAC a échoué et ont eu une césarienne non programmée Nous n’avons pas relevé de différence statistiquement significative entre le taux d’échec de la TVBAC dans le groupe de femmes d’un âge ≥35 ans (16,8%) par rapport à celles âgées de <35 ans (17,5%), p=0,86. Pour le groupe succès de la TVBAC, La durée moyenne du travail des patientes ayant eu une TVBAC avec succès était de 4,2 heures avec une durée moyenne de la phase latente de 4 heures et de la phase active de 2,2 heures. 64 accouchements par voie basse parmi les 350 se sont terminés par une extraction instrumentale soit dans 18,28 % des cas de succès de la TVBAC. Quatorze déchirures périnéales ont eu lieu dans ce groupe de parturientes, dont trois étaient de deuxième degré et deux de troisième degré. Pour le groupe échec de la TVBAC, le Tableau 2 résume les différentes indications de l’interruption de la TVBAC et le recours à une CS en urgence. Nous avons relevé quatre hémorragies du postpartum (HPP). Au total, nous avons relevé sept cas de ruptures utérines (RU) : cinq découvertes après l’accouchement par voie basse et deux au décours de la césarienne en urgence, ce qui correspondait à un taux de 1,7%. La révision utérine au décours d’un accouchement par voie basse après césarienne (AVBAC) était de réalisation systématique dans notre service afin de vérifier la vacuité utérine et surtout l’état de la cicatrice utérine.

**Tableau 1:** Répartition des parturientes selon la présence ou non d’un antécédent d’accouchement par voie basse après la césarienne

Antécédent d'accouchement par voie basse après la césarienne	Fréquence	Pourcentage(%)
Oui	74	17,5
Non	318	75,2
Donnée manquante	31	7,3
Total	423	100

**Tableau 2:** Les différentes indications de l’interruption de la TVBAC et le recours à une CS en urgence

	Nombre de cas	Pourcentage (%)
Stagnation de la dilatation	24	32,88
Souffrance fœtale aigue	15	20,55
Dystocie de démarrage	18	24,66
Défaut d'engagement	12	16,44
Suspicion d’une rupture utérine	3	4,1
Choriamniotite	1	1,37
Total	73	100

Nous avons pu détecter trois RU réparées chirurgicalement et quatre déhiscences utérines respectées car asymptomatiques. Deux des cinq RU après AVB ont été découvertes lors d’une exploration par laparotomie au bloc opératoire pour HPP immédiate avec examen sous valves et révision utérine trouvée sans anomalies. Le mode d’accouchement n’influençait pas d’une façon significative le risque de survenue d’une rupture utérine (groupe 1 :1,4% vs groupe 2: 1? 7% avec p=0,42). Nous n’avons pas eu recours à l’hystérectomie d’hémostase et ceci est valable pour les deux groupes. Le taux des transfusions sanguines en post-partum était de 1,4%. Nous n’avons observé aucune mortalité maternelle dans l’échantillon étudié. La comparaison des pourcentages de complications maternelles en fonction du mode d’accouchement effectif est rapportée dans le Tableau 3. Trois cas de mortalité périnatale ont été observés sur la totalité de notre échantillon: un dans le groupe 2 et deux dans le groupe 1.

**Tableau 3:** Comparaison des pourcentages de complications maternelles en fonction du mode d’accouchement effectif

Complications maternelles	Groupe 1(n=350)	%	Groupe 2(n=73)	%	P
Rupture utérine	5	1,43	2	2,74	
Saignement du post-partum	5	1,43	1	1,36	
Transfusion sanguine	6	1,71	0	0	
Déchirure périnéale	14	4	0	0	
Déhiscence utérine	3	0,85	1	1,36	
Hospitalisation prolongée	2	0,57	1	1,36	
Total	35	9,99	5	6,82	0,4

### ATCD d’AVB

Nous avons pu démontrer l’existence d’un lien entre l’absence d’antécédent d’AVB, particulièrement après une CS antérieure, et l’issue d’une TVBAC. Dans notre série 81% des parturientes n’ont pas accouché par voie basse après leur CS. Il existe une différence statistiquement significative (p=0,005) entre le taux d’échec de la TVBAC en cas d’absence d’antécédent d’AVB après la première césarienne (21% ) et sa valeur en cas de présence d’au moins un AVB après la première césarienne (7%) : OR=3,61 ; IC95%: 1,4 - 9,31 (Tableau 4 et Tableau 5). L’**intervalle inter génésique** le plus court dans notre série était de 11 mois et concernait un seul cas. 26 patientes dans le groupe 1 avaient un intervalle intergénésiaue de moins de 18 mois contre 5 dansle groupe 2. Ce facteur ne s’avère pas être associé à un échec significativement plus important (p=0,459).

**Tableau 4:** Mode d’accouchement selon l’indication de la césarienne antérieure

Indications des césariennes antérieures	Nombre	Groupe 1	Groupe 2	P
Antécédent d'une dystocie de démarrage	13	10(76,92%)	3(23,08%)	0,72
Antécédent d'une souffrance fœtale aigue	68	49(72,06%)	19(27,94%)	0,058
Antécédent d'un défaut d'engagement	9	3 (33,33%)	6(66,67%)	0,002
Antécédent d'échec de déclenchement	18	16 (88,88%)	2(11,12%)	0,54
Antécédent de stagnation de la dilatation	41	28 (68,29%)	13(31,71%)	0,049

**Tableau 5:** Mode d’accouchement selon la présence ou non d’un antécédent d’accouchement par voie basse après césarienne

Antécédent d'accouchement par voie basse	Groupe 1	Groupe 2	Total	p=0,005
Absent	252 (79,25%)	66(20,75%)	318(100%)	**OR=3,614**
Présent	69(93,24%)	5(6,76%)	74(100%)	
Total	321(81,89%)	71(18,11%)	392(100%)	

### Mode d’accouchement selon l’opérateur de l’échographie obstétricale et selon le poids fœtal estimé (PFE)

L’opérateur de la dernière échographie avant la TVBAC, qu’il s’agissait d’un résident en gynécologie-obstétrique ou d’un sénior (p=0,658) (Tableau 6), ne semblait pas être un facteur déterminant l’issue de la TVBAC. De même, le PFE entre 4000g et 4500g ne semblait pas influencer de manière significative le mode d’accouchement (p=0,166).

**Tableau 6:** Mode d’accouchement selon l’opérateur de l’échographie obstétricale

Opérateur d'échographie	Groupe 1	Groupe 2	Total	
Résident	151(80,75%)	36(19,25%)	187(100%)	**p=0,658**
Sénior	96(78,69%)	26(21,31%)	122(100%)	
Total	247(79,94%)	62(20,06%)	309(100%)	

### Issue de la TVBAC en cas d’un terme d’accouchement avancé

Nous avons étudié la relation statistique entre d’une part un terme gestationnel ≥40 SA et l’échec. Il existait une différence statistiquement significative entre le pourcentage d’échec en cas de terme d’accouchement ≥40 SA (21,03%) et le pourcentage d’échec en cas d’accouchement avant ce terme (13,66%). L’odds ratio était de 1,68 avec IC 95% : 1,01-2,8.

### Age des parturientes

Nous n’avons pas constaté de différence statistiquement significative entre le taux d’échec dans le groupe de femmes d’âge ≥35 ans par rapport à celles d’âge <35 ans (p=0,861).

### Hauteur utérine

Une hauteur utérine augmentée à terme (>33 cm) n’était pas associée à une augmentation statistiquement significative du taux d’échec de la TVBAC (p=0,169).

### Parité

Il existe une différence statistiquement significative entre le taux d’échec dans le groupe de parturientes ayant une parité <3 (22,99%) et celui des parturientes à parité ≥ 3 (8,02%) avec p<0,01. OR =3,42 ; IC95% :1,81- 6,46.

**Examinateur au début du travail:** nous avons constaté une différence statistiquement significative entre le taux d’échec de la TVBAC chez les parturientes examinées par un résident au début de travail comparées à celles examinées par un sénior avec une valeur de p<0,01. OR = 2,87; IC95% : 1,64 -5,1.

### Examen obstétrical

Des conditions obstétricales défavorables à l’entrée au box d’accouchement avec un score de Bishop <6 constituait un facteur statistiquement lié et d’une façon significative à l’augmentation de la probabilité d’échec de la TVBAC avec un p<0,01. OR=92,4 ; IC95%: 38- 225,3. La poche des eaux était intacte dans 67,6% des cas. L’état des membranes de la poche des eaux à l’admission en salle de travail n’a pas d’influence statistiquement significative sur l’issue de la TVBAC (p=0,508).

Le liquide amniotique était teinté à méconial dans 8,7% des cas et clair dans 91,3% des cas. Il n’y avait pas de différence statistiquement significative entre le taux d’échec en cas de liquide amniotique teinté et celui en cas de liquide amniotique clair (p=0,213). La présentation fœtale à l’admission en salle de travail était haute, refoulable dans 51,6% des cas et elle était appliquée à fixée à engagée dans 48,4% des cas, et l’échec de la TVBAC semblait être statistiquement indépendant du niveau de présentation du fœtus au niveau pelvien (p=0,155).

### Mode d’accouchement selon la durée du travail

Lorsque la phase de latence se prolongeait au-delà de six heures (groupe1 69,39% vs groupe 2 30,61%) la corrélation n’était pas statistiquement significative à un taux d’échec plus important de la TVBAC (p=0,132). Nous avons constaté d’après nos résultats statistiques que plus la durée de la phase active était courte plus les chances de succès de la TVBAC augmentaient, avec une valeur de p significative de 0,002. OR=4,26 ; IC95% : 1,57- 11,6. Elle a été calculée avec précision dans 391 cas parmi lesquels 17,14% ont connu un travail qui a duré plus que huit heures de temps et 82,86% avaient une durée totale de travail inférieure à 8 heures. Une durée de travail > 8heures était corrélée d’une façon significative à la TVBAC avec OR=2,44 ; IC95% : 1,34 - 4,5. Le mode d’accouchement selon la durée totale du travail est reporté dans le Tableau 7.

**Tableau 7:** Mode d’accouchement selon la durée du travail

Durée du travail	Groupe 1	Groupe2	Total	
>8h	47(70,15%)	20(29,85%)	67(100%)	p=0,0031
≤8h	276(85,19%)	48(14,81%)	324(100%)	OR=2,44
Total	323(82,6%)	68(11,4%)	391(100%)	

### RCF pathologique au cours du travail

Nous avons observé des interruptions de la TVBAC avec recours en urgence à la césarienne pour plusieurs types d’enregistrement cardiaque fœtal jugés anormaux : bradycardie fœtale, décélérations répétitives, décélérations DIP2, RCF micro-oscillant, RCF aréactif. Ces interprétations étaient corrélées à un taux d’échec statistiquement significatif des TVBAC avec OR=22,33 ; IC95% : 6- 82,6.

### Poids fœtal estimé et poids à la naissance

Nous avons constaté une macrosomie néonatale dans 9% des cas. Cette macrosomie n’était pas corrélée, d’après notre analyse statistique, à un risque supérieur d’échec de la TVBAC. Les 37 nouveaux nés avec un PN ≥ à 4000g avaient un PFE par échographie inférieur à 4000g, soit un taux de faux négatif de 8,8%. Par ailleurs, nous avons remarqué qu’il existait une sous estimation échographique statistiquement significative du poids fœtal pour les poids de plus de 3kg300 avec un T-test de 0,000.

## Discussion

### Facteurs historiques obstétricaux

### Antécédent d’accouchement par voie basse

Le taux d’échec de la tentative de voie basse en cas d’absence d’antécédent d’accouchement par voie vaginale après la première césarienne est plus élevé qu’en cas d’existence d’au moins un antécédent d’accouchement par voie vaginale après la première césarienne, comme l’ont montré les données de la cohorte multicentrique prospective de la MFMUN (Maternal-Fetal Medicine Units Network), rapportées par Grobman et al. en 2007, Landon et al. en 2005 [7] et Haumonté et al. en 2012 [8]. Ceci a été démontré également dans des études tunisiennes [9]. Dans notre série, cette différence était statistiquement significative (p=0,005).

### Intervalle inter génésique

A travers notre étude, nous n’avons pas retrouvé d’association entre ces deux entités. Ceci rejoint les études de de Landon et al. [**7**] et celle de Huang et al. [10] qui ne retrouvent aucune différence significative avec un délai de dix-huit mois et deux ans.

### Indication de l’ancienne césarienne

Les deux plus larges études prospectives de cohortes rapportées ont montré une diminution par deux du taux de succès de la TVBAC en cas de CS réalisée pour stagnation de la dilatation ou non-progression du travail par rapport à une autre indication de CS, notamment anomalie du RCF [7, 11]. Hoskins et al. [12] ont retrouvé un taux de succès de TVBAC de 67 % quand l’indication de la CS antérieure était une dystocie du travail avec dilatation cervicale de moins de 5 cm, 73 % en cas de non-progression du travail entre 6 et 9 cm et 13% en cas de non-descente de la présentation fœtale (p < 0,05). Notre étude rejoint la littérature : les indications comme une césarienne antérieure pour stagnation de la dilatation ou défaut d’engagement était associée à un taux d’échec statistiquement plus important de la TVBAC avec des OR respectifs de 2,056 (IC95% : 0,994 - 4,255) et 9 (IC95% : 2,19 – 37,37).

### Groupe d´âge des parturientes

L’étude de Shipp et al. [13] et celle de Bujold et al. [14] ont conclu toutes les deux à une réduction des chances d’accoucher par les voies naturelles avec l’augmentation de l’âge maternel. Cette constatation n’est pas spécifique à l’utérus cicatriciel [15]. Nous n’avons pas relevé de différence statistiquement significative entre le taux d’échec de la TVBAC dans le groupe de femmes d’un âge ≥35 ans (16,8%) par rapport à celles âgées de <35 ans (17,5%), p=0,86. Cette non-conformité aux résultats rapportés par la littérature pourrait être expliquée par le faible effectif de femmes d’âge avancé dans notre série : seulement 23 femmes étaient âgées de plus de 40 ans.

### Age gestationnel en début du travail

A travers notre travail, nous avons pu constater une augmentation du taux d’échec de la TVBAC en cas de terme d’accouchement ≥40 SA (21,03%) par rapport aux accouchements avant ce terme (13,66%) avec une différence statistiquement significative (p=0,046). Ceci rejoint les résultats de nombreuses études [8, 9, 16], montrant que les taux de succès de la TVBAC sont diminués en cas d’accouchement après 40 SA sans qu’il y ait de différence significative en ce qui concerne le risque de RU avant et après ce terme.

### Parité

Le CNGOF [17] a déclaré que la grande multiparité est associée à une réduction significative des échecs de la TVBAC et des risques de RU. La TVBAC devrait donc être encouragée pour les grandes multipares, il en est de même pour Kugler [18] et d’autres auteurs [19, 20]. Dans notre étude, il existait une diminution statistiquement significative du taux d’échec de la TVBAC dans le groupe des parturientes ayant une parité ≥3 (8,02%) comparé au taux d’échec chez les parturientes ayant une parité <3 (22,99%) et ce avec p<0,01.

### Hauteur utérine

L’évaluation de la hauteur utérine, comme outil clinique d’estimation du poids fœtal a été abordée par Adjahoto et Essafi [20, 21] et leurs équipes, montrant qu’une hauteur utérine > 34cm constituait un facteur de mauvais pronostic pour la TVBAC. Nous n’avons pas observé d’augmentation du taux d’échec en cas de hauteur utérine à terme >33cm.

### Déroulement du travail

Un score de Bishop favorable ou un col considéré comme favorable à l’entrée en salle de travail sont associés à une augmentation du taux de succès de la TVBAC [14, 22, 23]. Pour ce qui est de nos résultats, ils rejoignaient ceux décrits dans la littérature [24, 25]: un Bishop<6 ainsi qu’une présentation fœtale haute ou mobile étaient associés à des taux d’échec plus élevés de la TVBAC respectivement de (87,27%) et (22,56%) contre des taux de (6,91%) et de (16,23%) en cas de Bishop≥6 ou d’une présentation appliquée, fixée ou engagée respectivement. Ces différences étaient statistiquement significatives pour le facteur score de Bishop <0,01. La différence constatée en rapport avec le niveau de présentation fœtale n’était pas statistiquement significative (p= 0,155).

L’état de la poche des eaux à l’admission au début du travail ainsi que l’aspect du liquide amniotique à l’admission au début du travail n’ont pas eu beaucoup d’intérêt dans les études ceci rejoint nos résultats où la corrélation avec un échec de TVBAC n’était pas statistiquement significative. Pour la phase de latence, quand elle est prolongée au-delà de six heures, s’associait à un taux d’échec de la TVBAC plus important : 30,61% contre 20% dans notre série. Toutefois, cette différence n’était pas statistiquement significative (p=0,132) comme le montre Essafi [21]. Chez des patientes avec antécédent de CS, un arrêt de la dilatation cervicale en phase active était plus fréquemment retrouvé en cas de RU [23].

Essafi [21] a démontré à travers son étude que plus la phase active était rapide plus les chances de succès de la TVBAC étaient importantes. Dans notre étude, la durée de la phase active s’est avérée corrélée à l’issue de la TVBAC d’après notre étude: le taux d’échec de la TVBAC était de 36,84% en cas de phase active de durée ≥6 heures et de 12% quand elle a duré moins de 6 heures. Cette différence était significative avec p= 0,002. Lehmann et al. [22] n’ont pu identifier de lien entre la durée de travail et l’échec de la TVBAC. Dans notre étude, la durée totale du travail a été calculée avec précision dans 391 cas et une durée > 8heures était corrélée d’une façon significative à l’échec de la TVBAC avec p=0,0031.

### Poids fœtal estimé par l´échographie

Le facteur analysé dans la plupart des études était le PN, et non le poids fœtal estimé par échographie. Le CNGOF [17] a rappelé dans ses dernières recommandations que l’EPF par échographie reste très imprécise avec un risque d’environ 50 % de faux positifs pour prédire un PN supérieur à 4000g. Il n’existe aucune donnée en faveur d’une augmentation du risque de RU en cas d’une EPF échographique > 4000g : l’utilité de l’EPF échographique systématique n’a pas été démontrée [14]. Toutefois, un PN > 4500g ayant été associé à une augmentation modérée du risque de RU, une CPAC est alors recommandée si l’EPF échographique est > 4500g, particulièrement chez les patientes n’ayant pas accouché par voie vaginale [14]. Ceci rejoint les nombreuses études montrant que le nombre de RU n’étaient pas significativement augmenté en cas de macrosomie fœtale entre 4000g et 4500g [26–29]. La SOGC a déclaré en 2005 que la suspicion d’une macrosomie fœtale n’est pas une contre-indication à une TVBAC [30]. L’ACOG a considéré que l’ATCD d’AVB est un facteur protecteur contre la survenue d’une RU en cas de TVBAC avec macrosomie [31]. Dans notre étude, neufs fœtus ont été estimés macrosomes par échographie. L’issue de la TVBAC dans ces neuf cas était favorable.

### RCF pathologique au cours du travail

Lehmann a démontré à travers son étude qu’un rythme cardiaque fœtal pathologique est associé à une probabilité d’échec de la TVBAC augmentée [22]. Par ailleurs, a été étudié le lien entre les anomalies cardiaques fœtales et le diagnostic d’une RU. La RU survient dans 0,1 % à 0,5 % des UC et 0,2 % à 0,8 % des TVBAC. Les signes évocateurs d’une RU les plus fréquemment rapportés sont les anomalies du RCF associées ou non à une douleur pelvienne, d’apparition brutale et secondaire, mais ils ont une faible valeur diagnostique [17]. En cas de TVBAC, l’enregistrement en continu du rythme cardiaque fœtal est recommandé dès l’entrée en travail [17]. Dans notre étude, un RCF pathologique était corrélé à une augmentation du taux d’échec de la TVBAC: 78,5% contre 14,10% ; p=0,144.10^-9^.

### Poids néonatal

D’après le CNGOF, un PN > 4000g est associé à une augmentation minime à modérée du risque de RU. Toutefois, la TVBAC reste possible, compte tenu de son taux de succès élevé [17]. Un poids de naissance >4500g est associé à une augmentation modérée du risque de RU. Une CPAC est recommandée si de l’EPF échographique est>4500g particulièrement chez les patientes n’ayant pas accouché par voie vaginale auparavant [17]. Dans notre étude, le taux d’échec en cas de PN ≥4000g était plus important : 27% contre 16,19%. Cette différence n’était pas toutefois significative (p=0,094). En contre partie, nous avons constaté 37 nouveaux nés avec un poids supérieur ou égal à 4kg et dont le PFE était inférieur à 4000g, soit un taux de faux négatif de 8,8%. L’étude statistique de cette constatation nous a révélé qu’il existait une sous-estimation échographique statistiquement significative du poids fœtal pour les poids de plus de 3300g avec un T-test significatif de 0,000.

Cette différence des résultats entre l’échographie et le poids effectif à la naissance pourrait être expliquée d’une part par le caractère opérateur-dépendant de l’examen échographique et d’autre part par les conditions dans lesquelles se déroulaient parfois cet examen s’il est réalisé en début du travail : la non-coopération de la parturiente, la tête fœtale bas-située rendant le diamètre bipariétal difficile à mesurer, la quantité du liquide amniotique qui a éventuellement diminué et la circonférence abdominale comprimée et mal mesurée, tous ces facteurs peuvent rendre l’examen réalisé en urgence de mauvaise rentabilité et aboutir à une différence entre le poids estimé par échographie et le poids de naissance.

### Facteurs liés à l’organisation et à la prise en charge des parturientes au sein de l’établissement sanitaire

Peu d’études traitent des paramètres non médicaux et de leur susceptibilité d’être liés à l’issue de la TVBAC. L’hétérogénéité des méthodes de prise en charge, l’absence de consensus ou de recommandations et l’absence de protocoles écrits au sein des établissements sanitaires semblent être des facteurs limitant l’intérêt des auteurs à étudier ces paramètres. Au cours d’une enquête nationale sur la prise en charge et l’accouchement sur des UUC en France réalisée en 2009 [32], il a été constaté que 51% des maternités ne disposaient pas de protocole écrit de prise en charge des UUC. Le RANZCOG [33] a annoncé des recommandations claires en matière de prise en charge des parturientes avec UC en intra-partum: ces parturientes devraient être admises tôt dès le début du travail ; la surveillance du rythme cardiaque fœtal continue est impérative ; il faudrait que ces parturientes gardent le jeûne, devant la probabilité plus élevée d’avoir recours à une césarienne urgente; une estimation de la progression du travail par examen du col au moins 4 fois par heure a été recommandée. Le col devrait se dilater d’au moins 1 cm /heure au cours de la phase active. L’expulsion ne devrait pas excéder une heure de temps. Ces recommandations organisationnelles sont pratiquement les mêmes que celle adoptées par le CNGOF [17], le RCOG [34] et la HAS [35].

A travers notre étude, nous avons essayé d’évaluer le lien entre les conditions d’examen des patientes, leur examinateur, l’opérateur de l’échographie et l’issue de la TVBAC: nous avons constaté une différence statistiquement significative entre le taux d’échec de la TVBAC chez les parturientes examinées par un résident 15% au début de travail comparé à un examen réalisé par un sénior 34% (p<0,01). Cette différence pourrait être expliquée par le caractère en général plus lourd et jugé souvent plus à risque de complications ou d’échec, des cas examinés par un sénior. Il s’agissait donc d’un biais d’observation. Par contre, nous n’avons pas de différences significatives des taux d’échec selon que l’opérateur de l’échographie ait été un résident en gynécologie-obstétrique ou un sénior.

## Conclusion

Nos résultats sont rassurants concernant la TVBAC et ses issues dans notre service. Ils nous encouragent à continuer le développement de cette politique d’accouchement par les voies naturelles. Ceci devrait aller parallèlement avec une amélioration de nos structures sanitaires sur le plan humain et matériel. De cette façon, on pourrait proposer aux femmes le mode d’accouchement le plus naturel, donc le plus avantageux pour elle et son nouveau-né, dans un cadre de sécurité et de confiance réciproque. L’absence de protocole clair et écrit unifiant la prise en charge des utérus unicicatriciels et l’organisation de la TVBAC, a fait que de telles études, essayant d’étudier les facteurs de risque, soient sujettes à des biais de confusion.

Emettre donc un protocole aura des avantages multiples: rendre uniforme les conduites à tenir; clarifier les soins et les alternatives proposés aux patientes par l’établissement sanitaire en cas d’UC; faciliter la réalisation des enquêtes nationales et par conséquent l’établissement de recommandations nationales pour la prise en charge des utérus unicicatriciels.

La TVBAC, décidée après étude du cas rationnellement, revue des facteurs de risque d’échec et facteurs de bon pronostic, consentement de la patiente informée et éclairée, constitue une conduite sage et avantageuse pour la mère et son nouveau-né à court et à long terme et pour l’économie sanitaire de notre pays.

### Etat des connaissances actuelles sur le sujet

- Le taux de césarienne augmente partout dans le monde;
- Dans le cadre de la réduction du taux alarmant et croissant de césarienne recommandé par l’OMS, notre étude représente un modèle représentatif du succès de la voie basse dans l’épreuve utérine, en prenant en compte les différents facteurs de risque d’échec;
- Une surveillance maximale et continue est nécessaire pour diminuer la morbi-mortalité et représente une des recommandations de notre étude.

### Contribution de notre étude à la connaissance

- L’expérience d’un centre hospitalo-universitaire du nord-est tunisien;
- La tentative d’accouchement par voie basse sur utérus unicicatriciel nécessite des recommandations claires, notre étude recommande l’élaboration de guidelines nationaux adaptés à nos centres pour contribuer à réduire le taux de césarienne selon les recommandations de l’OMS, sans augmenter la morbi-mortalité materno fœtale.
